# Impact of GERAADA score in patients with acute type A aortic dissection

**DOI:** 10.1186/s13019-022-01858-y

**Published:** 2022-05-23

**Authors:** Kayo Sugiyama, Hirotaka Watanuki, Masato Tochii, Yasuhiro Futamura, Yuka Kitagawa, Satoshi Makino, Wataru Ohashi, Katsuhiko Matsuyama

**Affiliations:** 1 Department of Cardiac Surgery, Aichi Medical University Hospital, 1-1 Yazako Karimata, Nagakute, Aichi 480-1195 Japan; 2 Clinical Research Center, Aichi Medical University Hospital, 1-1 Yazako Karimata, Nagakute, Aichi 480-1195 Japan

**Keywords:** Acute aortic dissection type A, GERAADA score, Hemodialysis, Spouse presence

## Abstract

**Background:**

Despite continuous developments and advances in the perioperative management of patients suffering from acute aortic dissection type A (AADA), the associated postoperative morbidity and mortality remain high and strongly depend on the preoperative clinical status. The associated postoperative mortality is still hard to predict prior to the surgical procedure. The so-called German Registry of Acute Aortic Dissection Type A (GERAADA) score uses very basic and easily retrievable parameters and was specifically designed for predicting the 30-day mortality rate in patients undergoing surgery for AADA. This study evaluated impact of the GERAADA score in the authors’ institutional results.

**Methods:**

Among 101 acute type A aortic dissection patients treated at our hospital during August 2015–March 2021, the GERAADA was calculated individually and retrospectively. Predicted and actual mortalities were assessed, and independent predicted factors were searched. The primary endpoint was defined as comparison of GERAADA scores and early mortality, and the secondary endpoints were defined as comparison of GERAADA scores and other postoperative results, and comparison of preoperative factors and postoperative results regardless to GERAADA scores.

**Results:**

While the overall 30-day mortality for the entire study cohort calculated by the GERAADA score was 14.3 (8.1–77.6)%, the actual mortality rate was 6%. However, the GERAADA score was significantly high in some postoperative complications and showed significant correlation with some peri- and post-operative factors. In addition, factors not belonging to GERAADA score such as time from onset to arrival at the hospital, time from onset to arrival at the operation room, spouse presence, and hemodialysis were significantly associated with 30-day mortality.

**Conclusions:**

Although the actual mortality was lower than predicted, GERAADA score may impact on the postoperative course. In addition, it would be desirable to add parameters such as the time from onset to arrival, family background, and hemodialysis for further accuracy.

## Background

Acute aortic dissection type A (AADA) is a clinical emergency requiring early diagnosis and fast transfer to a specialized clinical center for open surgery as a definite treatment [1, 2]. Despite continuous improvements and developments regarding the perioperative strategies and surgical techniques, the associated 30-day mortality for AADA remains high and strongly depends on the preoperative clinical factors of the individual patient, such as the extent of dissection and presence of end-organ malperfusion [3–7].

In the management of AADA patients, survival is strongly dependent on a sufficient operative strategy and emergency surgery for AADA has become the gold standard, but the major influencing factors on mortality remain uncertain.

The GERAADA score is a simple effective tool to predict the 30-day mortality rate for patients undergoing surgery for AADA [8–10]. The main advantage of the GERAADA score lies in its simplicity. It may be calculated instantly either by the surgeon or a resident via a web-based calculator in almost any clinical situation once the basic parameters have been gathered by diagnostics, imaging, and physical examination of the patient at arrival. Luehr et al. also reported that the GERAADA score prediction of 30-day mortality after surgery is accurate, easily accessible due to its web-based platform and can be calculated with very basic preoperative clinical parameters [10].

However, in the GERAADA score, some important factors are not included. Although there has been little debate about hemodialysis because of its rarity, preoperative renal dysfunction has been reported to increase the operative risk of aortic repair for patients with AADA [11–13]. Although there are other influencing factors, timely diagnosis and treatment is essential for successful management in AADA [14, 15]. In addition, although there have been data describing that singles are prone to fatal myocardial infarction [16, 17], there have been few reports describing that marital status are related to outcomes of aortic dissection.

The aim of this study was to evaluate if the GERAADA score prediction corresponds with the authors’ institutional results and to identify the risk factors for mortality.

## Methods

### Study design

This single-center retrospective cohort study reviewed records of consecutive patients who underwent emergency surgical repair for AADA at Aichi Medical University Hospital from August 2015 to March 2021. Data collection comprised the 11 preoperative main parameters required for calculation of the new GERAADA score: age, sex, previous cardiac surgery, inotropic support at referral, resuscitation before surgery, aortic regurgitation (moderate to severe), preoperative hemiparesis, intubation/ ventilation at referral, preoperative organ malperfusion, extension of aortic dissection and location of primary entry site (Table 1). Other preoperative data not related to GERAADA score were also collected. Calculations of the GERAADA score were individually and retrospectively performed by a cardiac surgeon for all patients via a web-based application (https://www.dgthg.de/de/GERAADA_Score) [8–10]. The primary endpoint was defined as comparison of GERAADA scores and early mortality, and the secondary endpoint were defined as comparison of GERAADA scores and other postoperative results, and comparison of preoperative factors and postoperative results regardless to GERAADA scores.

**Table 1:** Characteristics of patients

	Number
*Factors in GERAADA score*	
Age > 75 (%)	26 (26)
Sex (male, %)	67 (66)
Previous cardiac surgery (%)	5 (5)
Resuscitation before surgery (%)	6 (6)
Intubation at referral (%)	6 (6)
Catecholamines at referral (%)	5 (5)
Aortic valve regurgitation (moderate to severe) (%)	22 (22)
Preoperative organ malperfusion (%)	41 (41)
Preoperative hemiparesis (%)	21 (21)
Extension of dissection, Asc: Arch: Des (%)	12 (12): 7 (7): 82 (81)
Location of primary entry tear within aortic arch, Asc: Arch: Des (%)	44 (44): 56 (55): 1 (1)
*Factors not in GERAADA score*	
Hypertension (%)	72 (71)
Smoking (%)	55 (54)
Chronic respiratory disease (%)	8 (8)
hemodialysis (%)	11 (11)
Coronary artery disease (%)	6 (6)
Cerebrovascular disease (%)	6 (6)
Family history (%)	3 (3)
Referral from other hospital (%)	59 (58)
Spouse or partner (%)	60 (59)
Cohabitant (%)	70 (69)
Time from onset to arrival at the hospital (minutes)	187 (24–1020)
Time from onset to arrival at the operating room (minutes)	291 (101–1257)

Asc, ascending aorta; Arch, Aortic arch; Des, descending aorta; GERAADA, German Registry for Acute Aortic Dissection Type A

### Study population

Diagnosis of AADA was confirmed by using contrast-enhanced computed tomography (CT) in our hospital or other previous hospitals. Preoperative CT imaging was used to determine the extent of the dissection, the location of the entry tear and potential organ malperfusion. Organ malperfusion, except for renal malperfusion, was defined due to evident clinical symptoms of coronary, cerebral, visceral, spinal, and peripheral malperfusion. Other data on patient demographics and comorbidities (hypertension, smoking history, chronic respiratory disease, chronic renal disease requiring hemodialysis, history of coronary artery disease, history of cerebrovascular disease, family background, and family history related to aortic dissection) were recorded. Chronic respiratory disease was defined as chronic obstructive lung disease or pulmonary fibrosis requiring specific medication. Coronary artery disease was defined as a history of coronary revascularization. Cerebrovascular disease was defined as permanent neurologic dysfunction. As a family background, patients were asked whether they had spouse or partner and whether they lived with family.

Both early and late clinical outcomes were assessed. Perioperative parameters, including time from onset to arrival at the hospital, time from onset to arrival at the operation room, operation time, cardiopulmonary bypass time, aortic cross clamp time, cerebral perfusion time, circulatory arrest time, and minimal body temperature, were assessed. Early outcomes included early mortality, cause of death, and other complications during hospitalization. Respiratory complications were defined as long-term ventilator dependence that required tracheostomy. Late outcomes included late mortality, cause of death, major adverse cardiac or cerebrovascular events (MACCE), and major adverse aortic events (MAAE). MACCE were defined as the composite of total death; myocardial infarction; stroke, hospitalization because of heart failure; and revascularization, including percutaneous coronary intervention, cerebrovascular intervention, and coronary artery bypass grafting. MAAE comprised a composite of either major aortic events or major aortic re-intervention. Major aortic events included rupture or re-dissection of the aorta. Major aortic re-interventions included additional thoracic endovascular aortic repair or a major surgical graft revision.

### Central repair procedure

Central aortic repair was performed for entry exclusion and true lumen reinstallation. The extent of graft replacement was based on the position of the primary entry. When the entry was in the ascending aorta, hemiarch replacement was selected, and when the entry was in the aortic arch, total arch or partial arch replacement was selected. An intra-bladder or pharyngeal temperature of 25 °C was set as the target. Cardiopulmonary bypass was basically instituted via bicaval drainage and both femoral artery and right axillary artery cannulation. Basically, conventional selective cerebral perfusion was initiated for brain perfusion.

### Statistical procedures

Continuous variables are expressed as mean ± SD or median (range), and categorical variables are expressed as the number (%) of patients. Categorical variables were analyzed using Fisher’s exact test. Continuous variables were compared using the Student’s t-test, whereas nonparametric variables were analyzed using the Mann–Whitney U-test. All data analyses were performed with the JMP 14.1 software (SAS Institute, Cary, NC, USA). Statistical significance was defined as *p* < 0.05.

### Definitions

All procedures were performed according to the tenets of the Helsinki Declaration, and the Ethics Committee of Aichi Medical University Hospital approved the study on September 28th, 2021 (approval number, 2021-412). All patients provided written consent for their clinical data to be used for scientific presentations or publications. The authors’ institution did not participate in the German Registry for Acute Aortic Dissection Type A (GERAADA).

## Results

The patients’ characteristics and preoperative data are summarized in Table 1. A total of 101 patients were admitted for AADA involving the ascending aorta. As factors related to GERAADA score, the mean age was 69 years (range, 34–88 years); 67 patients were male (66%). Previous cardiac surgery had been performed in 5 (5%) patients. At referral, 6 (6%) patients were already mechanically ventilated. Prior to surgery, inotropic support due to hemodynamic instability was required in 5 (5%) cases, while 6 (6%) patients had at least one episode of cardiopulmonary resuscitation since AADA onset. Six patients (6%) needed resuscitation owing to cardiopulmonary arrest because of coronary malperfusion (1 patient), cardiac tamponade (4 patients), and unknown (1 patient). Preoperative moderate to severe aortic regurgitation and end-organ malperfusion syndrome were diagnosed in 22 (22%) and 41 (41%) patients, respectively. Hemiparesis was evident in 21 (21%) patients. As factors not related to GERAADA score, eleven (11%) patients required hemodialysis and 59 (58%) patients were transported from other hospital. Sixty (59%) patients had spouse or partner and 70 (69%) patients lived with family. Time from onset to arrival at the hospital and time from onset to arrival at the operation room were 187 (24–1020) and 291 (101–1257) minutes.

Operative procedures and postoperative outcomes are summarized in Table 2. Early mortality occurred in six patients (6%) cases: three patients died of extensive cerebral infarction, one of descending aortic rupture, one of multiorgan failure, and one of coma. Late mortality occurred in four (4%) cases: two patients died of heart failure 3 months and 2 years after surgery and two patients died of unknown causes 3 months and 6 months after surgery. Follow up date was 295 (0–2022) days. During follow-up, MACCE occurred in 23 (23%) cases, in which nine patients died in the early and late period including one case of stroke, four patients needed redo open heart surgery, two patients needed percutaneous coronary intervention, one patient developed heart failure requiring hospitalization, and eight patients developed major cerebrovascular events. In redo open heart surgery, two patients underwent reanastomosis of artificial graft because of severe bending, one patient underwent aortic valve replacement, and one patient underwent aortic root repair. MAAE occurred in 19 (19%) cases, in which one patient died due to aortic rupture, four patients needed redo open surgery or open surgery for new aortic lesions, and fifteen patients underwent endovascular aortic repair or intervention for aortic disease including one case of redo open surgery.

**Table 2:** Operative procedures and outcomes

	Number
Central aortic repair (HAR, PAR, TAR)	48 (47.5): 9 (9): 44 (43.5)
Concomitant procedure	CABG 11, AVR 6, PVI 10, VSSR: 2
Operation time (minutes)	438 (241–865)
CPB (minutes)	237 (125–552)
ACC (minutes)	127 (82–362)
SCP (minutes)	112 (25–342)
CA (minutes)	51 (27–129)
ICU stay (days)	3 (0–45)
Hospitalization (days)	20 (0–98)
Cardiac event (%)	4 (4)
Neurological outcomes (%)	9 (9)
Tracheostomy (%)	6 (6)
Renal complication (%)	0
Transfer to other hospital	29 (20)
Early mortality (%)	6 (6)
Late mortality (%)	4 (4)
MACCE (%)	23 (23)
MAAE (%)	19 (19)

ACC, aortic cross clamp; AVR, aortic valve replacement; CA, circulatory arrest; CABG, coronary artery bypass grafting; CPB, cardiopulmonary bypass time; HAR, hemiarch replacement; ICU, intensive care unit; MAAE, major adverse aortic events; MACCE, major adverse cardiac or cerebrovascular events; PAR, partial arch replacement; PVI, pulmonary venous isolation; SCP, selective cerebral perfusion; TAR, total arch replacement; VSSR, valve-sparing aortic root replacement

**Fig. 1:**
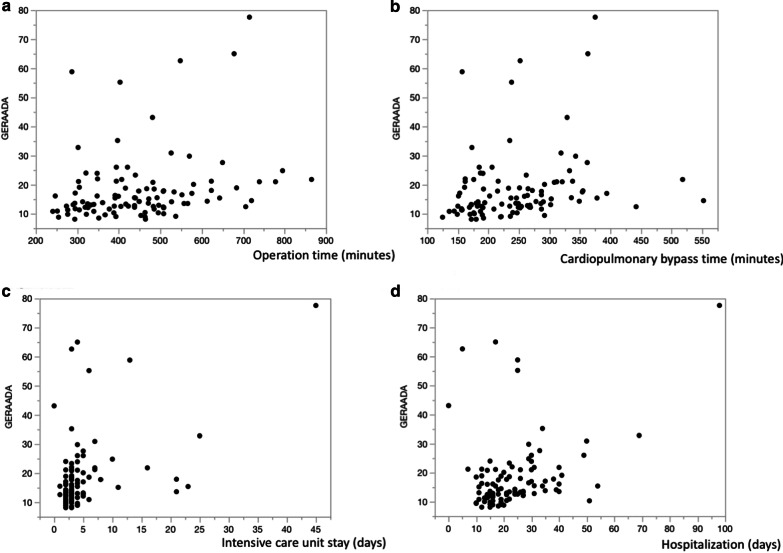
Postoperative factors and GERAADA score. **a** Operation time and GERAADA score. **b** Cardiopulmonary bypass time and GERAADA score. **c** Intensive care unit stay and GERAADA score. **d** Hospitalization and GERAADA score

**Fig. 2:**
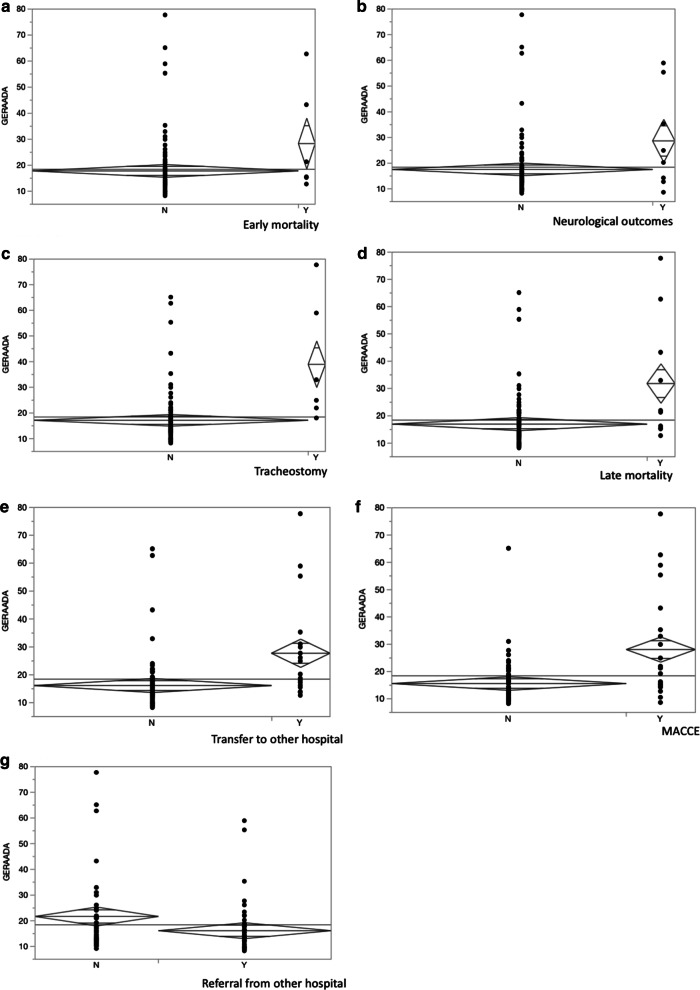
Preoperative and postoperative factors and GERAADA score. **a** Early mortality and GERAADA score. **b** Neurological outcomes and GERAADA score. **c** Tracheostomy and GERAADA score. **d** Late mortality and GERAADA score. **e** Transfer to other hospital and GERAADA score. **f** MACCE and GERAADA score. **g** Referral from other hospital and GERAADA score

GERAADA scores and postoperative results are compared. The overall 30-day mortality for the entire study cohort was calculated by the GERAADA score to be as high as 14.3% (range, 8.1–77.6), in comparison, the actual 30-day mortality rate of the study cohort was 6%. Due to the low mortality, it was difficult to evaluate the accurate area under the curve by receiver operating characteristic curve. However, the GERAADA score showed significant correlation with the following factors: operation time (*p* = 0.0081), cardiopulmonary bypass time (*p* = 0.018), intensive care unit stay (*p* < 0.0001), and hospital days (*p* = 0.0004) (Fig. 1a–d). In addition, the GERAADA score was significantly high in the following complications: early mortality (*p* = 0.041), neurological outcomes (*p* = 0.013), tracheostomy (*p* < 0.0001), late mortality (*p* = 0.0002), transfer to other hospital (*p* < 0.0001), and MACCE (*p* < 0.0001) (Fig. 2a–f). However, the GERAADA score was significantly lower for patients referred from other hospitals (*p* = 0.024) (Fig. 2g).

Comparison of preoperative factors and early mortality are summarized in Table 3. Factors being significantly associated with 30-day mortality belonging to GERAADA score included resuscitation before surgery (*p* = 0.0034), intubation at referral (*p* = 0.0034), catecholamines at referral (*p* = 0.001), and aortic valve regurgitation (moderate to severe) (*p* = 0.006). Other factors belonging to GERAADA score (high age, male, previous cardiac surgery, preoperative organ malperfusion, preoperative hemiparesis, extension of dissection and location of primary entry tear) alone were not significantly associated with 30-day mortality. On the other hand, factors being significantly associated with 30-day mortality but not belonging to GERAADA score included time from onset to arrival at the hospital (*p* = 0.0079), time from onset to arrival at the operation room (*p* = 0.0009), not having spouse or partner (*p* = 0.026), and hemodialysis (*p* = 0.042). Other factors not belonging to GERAADA score (hypertension, smoking, chronic respiratory disease, coronary artery disease, cerebrovascular disease, family history, referral from other hospital and not having cohabitant) were not significantly associated with 30-day mortality.

**Table 3:** Preoperative factors in early mortality

	*p* value
*Factors in GERAADA score*	
Age > 75	0.42
Sex (male)	0.97
Previous cardiac surgery	0.17
Resuscitation before surgery	0.0034
Intubation at referral	0.0034
Catecholamines at referral	0.001
Aortic valve regurgitation (moderate to severe)	0.006
Preoperative organ malperfusion	0.18
Preoperative hemiparesis	0.069
Extension of dissection in Arch or Des	0.26
Location of primary entry tear within Arch or Des	0.16
*Factors not in GERAADA score*	
Hypertension	0.23
Smoking	0.54
Chronic respiratory disease	0.46
hemodialysis	0.042
Coronary artery disease	0.53
Cerebrovascular disease	0.64
Family history	0.66
Referral from other hospital	0.2
Spouse or partner	0.026
Cohabitant	0.31
Time from onset to arrival at the hospital > 300 min	0.0079
Time from onset to arrival at the operating room > 300 min	0.0009

Statistical significance was set at *p* < 0.05

Asc, ascending aorta; Arch, Aortic arch; Des, descending aorta; GERAADA, German Registry for Acute Aortic Dissection Type A

## Discussion

Acute aortic dissection type A (AADA) is a cardiovascular emergency and necessitates immediate diagnosis and subsequent surgical treatment to overcome the risk of preoperative deterioration and death [2, 5]. Further, the associated postoperative mortality is still hard to predict prior to the surgical procedure or when explaining the situation to the patient or to the patient’s relatives (e.g. if the patient is unconscious) in the emergency room. As risk factors associated with postoperative mortality, there have been many reports, however, it remains controversial.

In the present study, because the actual 30-day mortality rate of the study cohort was low (6%) compared to calculated GERAADA score (14.3%), it was difficult to evaluate the accurate area under the curve by receiver operating characteristic curve. Conzelmann et al. reported that 16.9% died within 30 days [5]. Luehr et al. reported that actual and predicted 30-day mortality were 15.1% and 15.7% [10]. Mehta et al. reported that in-hospital death was 26.9% in surgically treated patients [12]. Goda et al. reported that 41 patients (13.6%) died during hospitalization [14]. Taking into consideration of these results as mentioned above, our mortality rate was satisfactory, but too low for statistical evaluation. In accordance with Luehr et al., the highest GERAADA scores were calculated for the following subgroups: resuscitation before surgery (52.0%), catecholamines at referral (41.8%), intubation at referral (37.1%) and coronary malperfusion (30.0%). In the present study, there was a significant association with 30-day mortality in resuscitation before surgery (*p* = 0.0034), intubation at referral (*p* = 0.0034), catecholamines at referral (*p* = 0.001), and aortic valve regurgitation (*p* = 0.006), and these calculated GERAADA score were as follows; resuscitation before surgery (55.8%), intubation at referral (60.4%), catecholamines at referral (61.4%), and aortic valve regurgitation (15.8%). The remaining three factors, except for aortic valve regurgitation, showed high predicted mortality compared to other factors related or not related to the GERAADA score. Further, the GERAADA score revealed significant correlation with peri- and post-operative factors. The GERAADA score was significantly high in the following complications: early mortality (*p* = 0.041), neurological outcomes (*p* = 0.013), tracheostomy (*p* < 0.0001), and late mortality (*p* = 0.0002). In addition, the GERAADA score showed significant correlation with the following factors: operation time (*p* = 0.0081), cardiopulmonary bypass time (*p* = 0.018), aortic cross clamp time (*p* = 0.071), intensive care unit stay (*p* < 0.0001), hospital days (*p* = 0.0004), transfer to other hospital (*p* < 0.0001), and MACCE (*p* < 0.0001). Therefore, GERAADA score may impact on the postoperative course. Czerny et al. stated that the GERAADA score is not meant as an apodictic instrument for accepting or rejecting treatment but is meant to serve as a useful instrument to be able to anticipate postoperative outcome according to very basic and easily retrievable parameters [8, 9]. Pollari et al. stated that the best use of the new GERAADA score should not be the patient selection or the decision-making but rather the quality control and the performance comparison between different hospitals for the retrospective evaluation and better resource management purposes [18]. For these reasons, The GERAADA score can be a useful predictor of other postoperative course as well as 30-day mortality.

In addition, as factors not belonging to GERAADA score, parameters such as the time from onset to arrival at the hospital, time from onset to arrival at the operation room, spouse presence, and hemodialysis were significantly associated with early mortality.

Goda et al. described renal dysfunction defined by a serum creatinine concentration of more than 2.0 mg/dL, was a preoperative independent risk factor for hospital mortality in AADA surgery [11]. According to Mehta et al., as variables predicting in-hospital death after AADA surgery, kidney failure was given the highest score [12]. As shown in these reports, preoperative renal dysfunction has been reported to increase the operative risk of aortic repair for patients with AADA, however, there have been little debate about hemodialysis. Akiyoshi et al. reported that the difference was not statistically significant between hemodialysis group and non-hemodialysis group although the in-hospital mortality rate was increased in hemodialysis group [13]. According to Akiyoshi et al., although in-hospital mortality was increased in dialysis group (21%) compared to non-dialysis group (7%), morbidities did not differ significantly. Since aortic dissection is somewhat rare in hemodialysis patients, further studies on the effects of hemodialysis on the outcome of AADA are warranted.

Not to mention, timely diagnosis is essential for successful management in AADA. Nakai et al. described that time from symptom onset to operation within 5 h is a significant predictor of long-term survival among patients with AADA and preoperative malperfusion [14]. Nakai et al. reported that there was no significant difference in the early mortality rates between patients with immediate (transferred to the operation room within 300 min of the onset of symptoms) and later aortic repair, which were 20.0% and 26.9%, respectively [14]. However, the cumulative 5-year survivals of patients with malperfusion syndrome in the immediate and later repair groups were 76.7% and 45.4%, which was a significant difference [14]. According to Harris et al., a median time to diagnosis of 4.3 h plus an additional 4.3 h from diagnosis to surgery indicates that there is a significant opportunity for systematic improvement [15]. The authors reported that some patient groups, such as patients who transfer from other hospitals, those without pain, nonwhites, and those with prior cardiac surgery, are prone to delays in treatment [15]. In the present study, time from onset to arrival at the hospital and time from onset to arrival at the operation room were significantly associated with 30-day mortality. Moreover, interestingly, those related with lower GERAADA scores were referred patients from other hospitals. This indicates that critically ill patients are not referred or transported in time, and it is the relatively mildly ill that are transported. Harris et al. also described that patients who transfer from primary hospitals was related to the greatest delay in time to surgery [15]. In order to save time, it is critical to establish acute aortic syndrome network consisting experienced aortic centers as well as prompt diagnosis with advanced imaging technology. In addition, we endorse the crucial role of modern dedicated specialized aortic centers in treating acute aortic syndromes.

Although there have been no reports about relationship between aortic dissection and family background, the relationship between coronary artery disease and family background has been described [16, 17]. In the present study, patients without a spouse were more associated with early mortality (*p* = 0.026). Single men and women with myocardial infarction reportedly had an increased mortality compared with married participants [16, 17]. Although the causal relationship is still unclear, there have been data describing that singles are prone to fatal myocardial infarction. Fournier et al. suggested that having information of ‘living alone’ versus ‘living with someone’ instead of married versus not married would have been more useful in cardiovascular events [19]. On the other hand, in the present study, patients without a cohabitant were not associated with early mortality (*p* = 0.31). Further studies on the relationship between AADA and family background are warranted.

This study is had some limitations. First, relatively few patients were included owing to the rarity of this condition. Second, this was a retrospective single-center experience lacking any form of randomization. Some figures contain some outliers, which may have made accurate statistical evaluation difficult. Third, the surgical technique for AADA has evolved during the time of this study. To resolve these limitations, a multi-institutional study is needed. Further, a prospective clinical trial is required to further evaluate the new GERAADA score as a useful tool to allow for improved decision- making in the emergency setting of AADA. Moreover, public-health management strategies that would reduce the time from onset to admission are needed.

## Conclusions

Although the actual early mortality was lower than predicted, the presented results demonstrate the effectiveness of the GERAADA score on postoperative course. In addition, it would be desirable to research and add parameters such as the time from onset to arrival, spouse presence, and hemodialysis for further accuration.

## Data Availability

Not applicable.

## Abbreviations

AADA: Acute aortic dissection type A
CT: Computed tomography
GERAADA: German Registry for Acute Aortic Dissection Type A
MAAE: Major adverse aortic events
MACCE: Major adverse cardiac or cerebrovascular events
